# News and Miscellany

**Published:** 1884-01

**Authors:** 


### NEWS AND MISCELLANY.

Tuberculosis in Wild Animals.—A statement is made in the "Popular Science Monthly," to the effect that among animals in the wild state consumption is much more prevalent than among those in captivity, owing to exposure and to the impossibility of the beasts escaping in their lairs from the effects of violent storms.

Prolonged Lactation in a Cow.—Saint-Ives Ménard, subdirector of the Acclimatation Garden in Paris, reports the case of a spayed cow in which the lacteal secretion persisted three years and eight months, during which time she gave 12,600 litres of milk rich in solid constituents. According to Ménard the milk of spayed cows is always much superior in quality to that of the non-spayed ones.

Heroic Treatment.—An elephant attached to a commissáriat depôt, was suffering from constipation of the bowels, and no veterinary surgeon being with the troop, the officer in charge was puzzled as to what treatment to adopt. An enema was suggested; the next thing was how to administer it.

No other way out of the difficulty presenting itself, a detachment was ordered out with the fire engine, and an enormous quantity of water was injected into the suffering animal.

The elephant died; but whether from the disease or the remedy adopted will never be known to science, as no *post-mortem* examination was made.—*The Veterinary Journal.*

Rheumatism in Cattle.—There is something remarkable in the tendency to rheumatic affections so frequently observed in the ligaments and synovial membranes of the joints of cattle, and likewise in the *fascia* or celluar coat of the muscles. The disease is attended by stiffness and inability to move; pain on pressure, and more or less febrile symptons Sometimes it attacks one or more of the joints, and occasionally shifts its action to the others. This tendency of the disease to shift from one part to another, is evidence of constitutional affection, and dependent on temperment and state of the circulating fluids. Among the causes which predispose to rheumatism, must be placed an hereditary tendency and temperment of the animal, for although we find it prevalent in cold, marshy districts, in exposed places, and during the spring and autumn months, when there is the greatest vicissitude of heat and cold, yet, why the same agents should produce rheumatism in one case, bronchitis in another, pleurisy in a third, and dysentery in a fourth, and so on, can only be explained by supposing that each individual has some particular organ or organs which are more susceptible to disease than other parts of the organization.—*National Live-Stock Journal, Chicago.*

Shoulder Lameness.—Shoulder lameness of horses is not of nearly so frequent occurrence as is generally imagined; but sometimes the difficulty in ascertaining the real seat of lameness, when situated in the foot, has occasioned many an ignorant smith to refer the complaint to the shoulder; and the poor animal has in consequences been doomed to undergo the painful operations of blistering, firing and rowelling. It is of considerable importance, therefore, to be able to distinguish sprains of the shoulders from other ailments. Mistakes will seldom occur if attention be paid to the following symptons: When a horse is lame in the shoulder he drags his toe along the ground, from inability of the muscles of the shoulder to lift the foot from the ground. If he lifts his foot high, the shoulder cannot be much affected. On walking down hill, he catches up the leg with considerable quickness. He will frequently stumble on going up hill, and will make a shorter step with the lame leg than with the other. He goes equally lame on soft or hard ground, which is not the case when the lameness is in the foot. In shoulder lameness there is no difference in the temperature of the two fore feet.—*Breeder's Gazette, Chicago.*

